# Effect of sodium dodecyl sulfate on the production of L-isoleucine by the fermentation of *Corynebacterium glutamicum*

**DOI:** 10.1080/21655979.2020.1831364

**Published:** 2020-10-21

**Authors:** Haibo Xiong, Yunpeng Liu, Qingyang Xu

**Affiliations:** a College of Biological Engineering, Tianjin University of Science and Technology, Tianjin, China; b National and Local Joint Engineering Laboratory of Metabolic Control Fermentation Technology, Tianjin, China

**Keywords:** Fermentation optimization, cell membrane, ‘fermentation conversion’, threonine dehydrase, acetohydroxy acid synthase, metabolic flow analysis

## Abstract

*Corynebacterium glutamicum* is a safe and popular industrial microorganism that it is gram-positive bacteria with thick cell walls, which hinder the extracellular secretion of products. Surfactant has good surface or interface activity and can destroy the cell membrane of microorganisms. In this study, the surfactant SDS was used to artificially destroy the cell membrane of *Corynebacterium glutamicum*, increase the permeability of the cell membrane, and increase the ability of the strain to secrete L-isoleucine. This is the first time that surfactants have been applied to the fermentation of *Corynebacterium glutamicum*. Results indicated that after optimization, the output of L-isoleucine reached 43.67 g/L, which was 13.01% higher than that without sodium dodecyl sulfate. The yield of the by-products, such as valine, leucine, and alanine, was reduced by 72.30%, 64.30%, 71.70%, respectively. This method can promote the production of L-isoleucine while minimizing the damage of SDS to the strain.

## Graphical abstract

**Figure:**
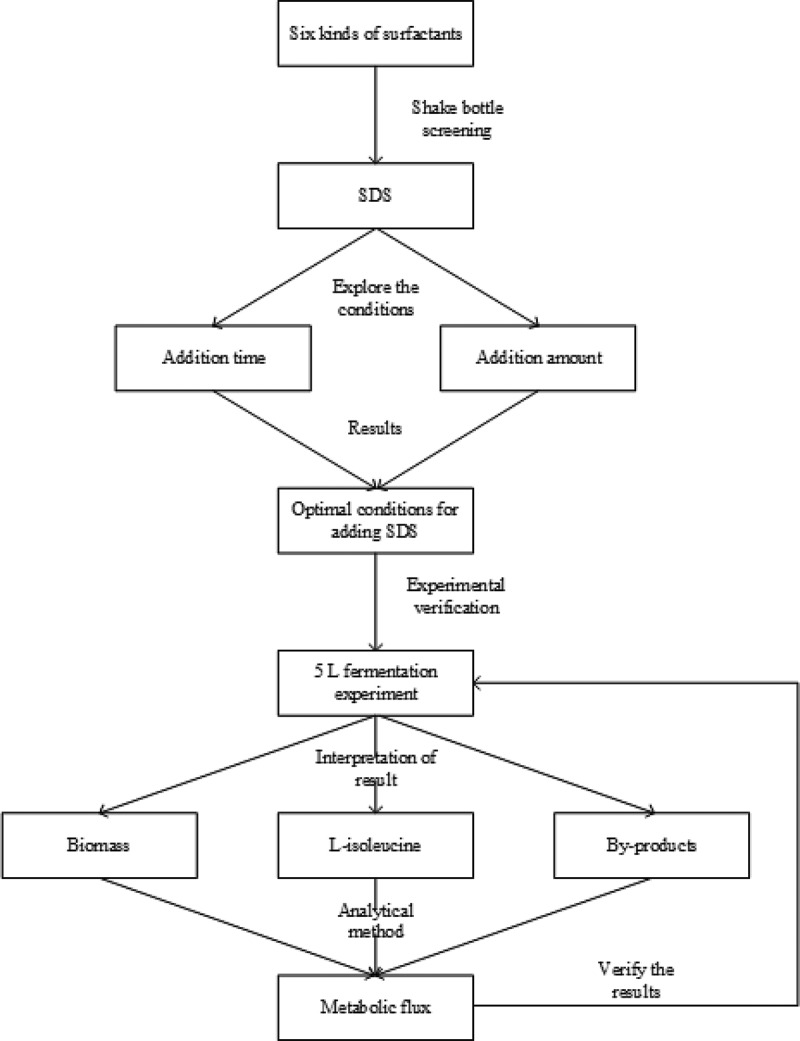

## 1 Introduction

L-isoleucine is an essential amino acid for the human body and one of the three branched-chain amino acids[1]. Dou to its special structure and function, it occupies a particularly important position in human life metabolism. L-isoleucine, one of the more expensive amino acid raw materials, is mainly made into compound amino acid infusion, three-branched-chain amino acid infusion, which aims to treat liver disease, liver coma, frailty, and other diseases [2,3]. The industrial production process of L-isoleucine is mainly microbial fermentation that can realize large-scale industrial production, and reduce the cost of raw materials and easy to control reaction, but at the same time, large amounts of by-products are produced (such as valine, leucine, and alanine) [4]. L-isoleucine is mainly used in the food and medicine fields, which needs the high purity of the product, and thereby excessive by-products are not conducive to subsequent extraction and purification [5].

Surfactants are a kind of substances that can significantly reduce the surface (interface) tension even at very low concentrations [6,7]. In previous reports, surfactants were used in kitasamycin fermentation and the fermentation of waste-activated sludge [8]. Sodium dodecyl sulfate (SDS) is an anionic surfactant, which stimulates microbial fermentation and enzymatic activity in bioreactors, and also destroys microbial cell membranes [9].

In this study, the surfactant was applied to the fermentation of *Corynebacterium glutamicum* for the first time. The use of surfactants to interfere or destroy the forming or the formed cell membrane to achieve the effect of enhancing the permeability of the cell membrane [9]. Enhanced cell membrane permeability relieved the inhibition of the end product produced by the excessively high intracellular product concentration, improved the secretion ability of L-isoleucine, meanwhile reduced the by-product production [10].

## 2 Materials and methods

### 2.1 Strain

The *Corynebacterium glutamicum* (The strain number is YILM1504) (Tianjin University of Science, Tianjin, China) used was preserved by the Metabolic Engineering Laboratory of Tianjin University of Science and Technology.

### 2.2 Fermentation medium

The fermentation medium consisted of 70 g/L glucose, 10 g/L peptone, 2 g/L yeast powder, 3 g/L (NH_4_)_2_SO_4_ · 7H_2_0, 2.2 g/L KH_2_PO_4_, 0.2 mg/L vitamin B, 20 mL/L corn hydrolyzate, 1 g/L lysine, 3 g/L glutamic acid, 0.2 g/L methionine, 50 g/L corn steep liquor. All chemical reagents were of analytical grade and were produced by Aladdin (Shanghai Alighting Biochemical Technology Co., LTD, Shanghai, China).

The Surfactants used in this experiment are Tripion-X, SDS, Sophorolipid, Surfaction, NP-10, Tween-80, all chemical reagents were of analytical grade, and were produced by Deyan (Shandong Deyan Chemical Co. LTD. Shandong, China).

### 2.3 Experimental methods

The preservation tube by *Corynebacterium glutamicum YILM 1504* was removed from the refrigerator at −80°C (DW-45/60/86L550 vertical ultra-low temperature storage box, Jiesheng Refrigeration, Zhejiang, China), which transferred to two oblique interview tubes in sterile intermittent culture. It is then activated in a 32°C incubator for 24 h, and transferred 200 ml each of the activated strain to two Eggplant-shaped flasks were cultured at 32°C for 24 h to maintain vigorous strains.

3 L seed culture medium was placed in a 5 L fermenter (5 L automatic control fermentation tank, Shanghai Baoxing Biological Equipment Engineering Co., Ltd., Shanghai, China) and the seed cultivation time was 16 h at 32°C, and maintained at pH 6.7–7.0. In addition, the initial ventilation, tank pressure, and rotation speed was 2.0 L/min, less than 0.05 MPa, and 200 r/min, respectively. During this period, the rotation speed was gradually adjusted to 400 r/min according to oxygen flux in the fermenter, but other conditions remain unchanged. The OD_600_ (752 Spectrophotometer, Shanghai Analytical Instrument Factory, Shanghai, China) greater than 16 was connected to fermentation culture.

The 600 mL cultivated seed liquid was put into 3 L fermentation medium and was continuously cultured for 46 h at the maintenance temperature of 32°C and pH 6.7–7.0. Due to the need to keep dissolved oxygen was maintained between 30% and 40%, so the stirring shaft speed was gradually increased from 200 r/min to 900 r/min, and the ventilation was gradually increased from 2.0 L/min to 6.0 L/min. The residual sugar was measured after 16 h, and 80% (volume fraction) glucose was added to maintain the residual sugar in the tank between and 10–20 g/L.

In this experiment, three types of six surfactants were selected, and 1 g/L was added to each shake flask at the initial stage of fermentation. After 40 hours of fermentation, samples were taken to determine the strain biomass and L-isoleucine production in the fermentation broth.

### 2.4 Detection methods

#### 2.4.1 Strain biomass determination

Strain biomass was expressed as the dry weight of strain cells per liter of fermentation broth (g DCW/L) (ZA-RZ series electronic balance, Shanghai Zanwei Weighing Apparatus Co., Ltd., Shanghai, China). After the fermentation broth was centrifuged, the cells were washed twice with deionized water, heated to constant weight in a thermostat at 80°C, and weighed with an analytical balance.

#### 2.4.2 Determination of L-isoleucine and by-products

The concentration of L-isoleucine and by-products in the fermentation broth was determined by high-performance liquid chromatography using an Agilent C18 (15 mm×4.6 mm, 3.5 μm) chromatographic column, and the derivatizing agent was 2,4-dinitrofluorobenzene. Mobile phase was 50% acetonitrile, 4.1 g/L sodium acetate solution with the column temperature at 33°C, flow rate at 1 mL/min, and detection wavelength at 360 nm (Agilent1200, Agilent Technologies, California, U.S.).

#### 2.4.3 Determination of threonine dehydratase enzyme activity

Cells were harvested by centrifugation for 10 min at 8,000 r/min at 4°C and were washed the strain twice with 50 mmol/L Tris-HCl, and resuspend the strain. Then, the suspended strain cells were disrupted by ultrasonic (2 s on 3off 4°C 400 W 10 min), and centrifuged at 4°C 12,000 r/min for 30 min to remove cell debris. The supernatant was the crude enzyme solution.

A volume of 400 µL pyridoxal phosphate (2.5 mM) and 100 µL crude enzyme solution was added to a 5 mL Eppendorf tube sequentially, then 500 µL threonine (80 mM) was added to start the reaction and placed in a 22 ± 2°C water bath for 15 min. The reaction was terminated by adding 1 ml of a solution containing 1% semicarbazide and 0.9% sodium acetate, after 15 min of incubation at room temperature, the amounts of a-ketobutyrate formed at various times were determined spectrophotometrically by monitoring the semicarbazone derivative at 254 nm.

#### 2.4.4 Determination of acetohydroxy acid synthase enzyme activity

After adding 500 µL of sodium pyruvate (200 mM), 200 µL of thiamine pyrophosphate (1 mM), and 200 µL of MgCl_2_ (50 mM), 100 µL of crude enzyme solution to the Eppendorf tube to start the reaction, the solution was placed in a water bath at 37°C for 15 min. A total of 100 µL of 3 M H_2_SO_4_ was added to stop the reaction, then placed in a 62°C water bath for 15 min, and centrifuged at 12,000 rpm for 10 min. A total of 0.25 mL of the supernatant, 0.5 mL of creatine (0.5%), 0.5 mL of ɑ-naphthol (5%), and 2.25 mL of water was placed in a 5 mL Eppendorf tube and reacted in the dark at room temperature for 60 min, and then the OD value was measured with a 530 nm spectrophotometer.

### 2.5 *Establishment of a metabolic flow balance model of* C. glutamicum *producing L-isoleucine*

#### 2.5.1 Metabolic flux calculation

Through the establishment of a metabolic flow balance model for isoleucine biosynthesis, quantitative analysis and network optimization analysis of the metabolic network were carried out, and the metabolic flow distribution in the middle and late stages of L-isoleucine fed-batch fermentation was calculated, providing a basis for genetic manipulation and fermentation control of L-isoleucine producing strain.

Using the method of Joseph J.Vallino, the accumulation rate of metabolites calculated according to the material balance is:

$$r_{j}\left(t\right)=\sum a_{j}x_{j}\left(t\right)-\sum a_{k}x_{k}\left(t\right)$$

where $x_{j}\left(t\right)$ — reaction rate of the jth step reaction, mmol/L·h;

$x_{k}\left(t\right)$ — reaction rate of the kth step reaction, mmol/L·h;

$a_{j}$ — Reaction measurement coefficient of the jth step reaction;

$a_{k}$ — Reaction measurement coefficient of the kth step reaction;

$r_{j}\left(t\right)$ — accumulation rate of intermediate metabolite j, mmol/L·h;

From the pseudo-steady-state assumption, $r_{j}\left(t\right)=0$. The m intermediate metabolites in the metabolic network constitute n metabolic flow balance equations, written in matrix form as

$$Ax\left(t\right)=0$$

In the formula, A is αν μ × n-dimensional matrix, $x\left(t\right)$ is αν ν × 1-dimensional matrix, and n is the total number of selected rates. Degrees of freedom of the problem to be solved:

F=n−m

#### 2.5.2 Establishment of the metabolic network

*C. glutamicum* has several pathways including glycolysis (EMP), tricarboxylic acid cycle (TCA), and hexose monophosphate (HMP). The HMP can provide a large amount of NADPH for amino acid synthesis, which is of great significance in the synthesis of isoleucine. Thus, the complete pathway should be preserved in the construction of the isoleucine biosynthetic pathway. There are also various enzymes involved in the TCA replenishment reaction. However, studies have shown that when *C. glutamicum* is cultured in a medium with glucose as a substrate, the glyoxylic acid branch does not appear, which indicates that the TCA cycle is still the main oxidation pathway in the fermentation process of *C. glutamicum*.

The following are the principles of establishing a metabolic network: (1) cells are in a non-growth period or the cell concentration changes little and can be ignored; (2) there is no glyoxylic acid cycle in cell metabolism; (3) NADPH consumed in the reaction pathway, and the total number of NADPH produced by the HMP and TCA cycles is equal; (4) the total ATP balance is not considered; (5) and the reaction without branch nodes during the reaction is calculated as a one-step reaction.

### 2.6 Calculation methods

#### 2.6.1 Acid-to-mass ratio

The acid-to-mass ratio is as follows:

$$t\left(\%\right)=\frac{V_{1}}{V_{2}}\times 100\%$$

where t is the mass ratio of L-isoleucine produced per liter of fermentation broth to biomass after 40 h of fermentation, referred to as acid-to-mass ratio, %; V_1_ is the mass of L-isoleucine per liter of fermentation broth after 40 h of fermentation, g/L; and V_2_ is the strain biomass per liter of fermentation broth after 40 h fermentation, g/L.

#### 2.6.2 Sugar and acid conversion rate

The calculation formula of the conversion rate of glucose to L-isoleucine sugar acid SA_1_

$$SA_{1}/\%=\frac{\rho_{1}\times V_{1}}{m}\times 100$$

where $SA_{1}$ is conversion rate of glucose to L-isoleucine sugar acid, %; ρ_1_ is the mass concentration of L-isoleucine, g/L; V_1_ is the total volume of fermentation broth, L; and m is the total sugar consumption, g.

Calculation formula of the conversion rate SA_2_ of glucose to strain biomass

$$SA_{2}/\%=\frac{\rho_{2}\times V_{2}}{m}\times 100$$

where $SA_{2}$ is conversion rate of glucose to strain biomass, %; ρ_2_ is the cell mass concentration, g/L; V_2_ is the total volume of fermentation broth, L; and m is the total sugar consumption, g.

## 3 Results and discussion

### 3.1 Cultivation in shaker flasks to explore the best surfactant

It has been previously reported that surfactants can improve the permeability of cell membranes and are often used to increase the yield of target products in microbial fermentation. It can speed up material delivery and increase the intracellular and extracellular material balance.

**Figure 1.**
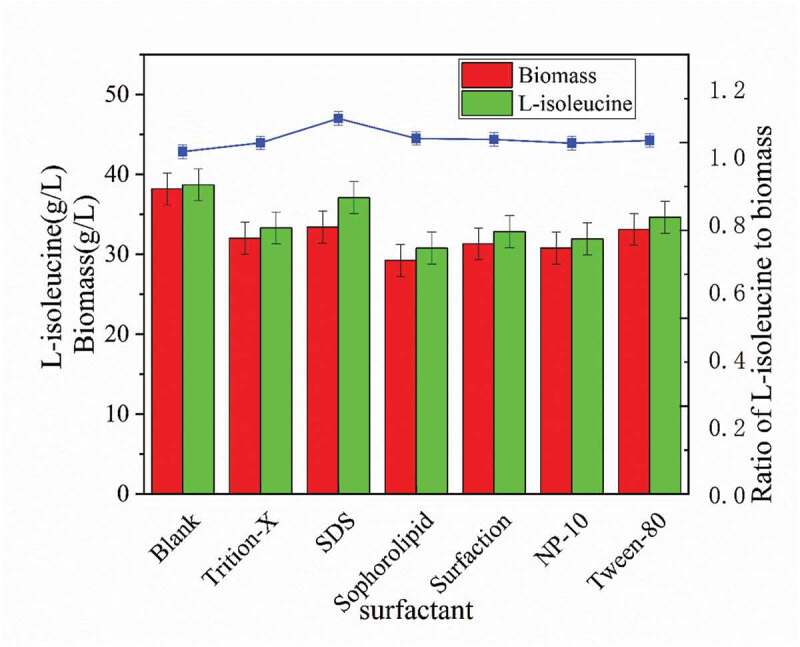
Effects of different surfactants on the biomass and acid production capacity of strain

As seen in Figure 1, surfactants had a significant inhibitory effect on strain growth, yet sodium dodecyl sulfate (SDS) had the least effect on strain growth. The biomass of strain cells and L-isoleucine production decreased by 12.59% and 4.19%, respectively. Moreover, comparing the ratio of L-isoleucine to the biomass (acid-to-mass ratio) curve produced by the strain, the addition of surfactants improved the acid-to-mass ratio of the strain [11]. The SDS had the highest increase in the acid-mass ratio, reached 1.11%, increased by 9.67% compared to the control group without addition. It can be clearly seen that surfactants are harmful to the growth of strain, which also leads to the decrease of L-isoleucine production. Because the production of L-isoleucine requires a lot of strain to work. However, according to the aspect of acid-to-mass ratio, surfactants increased the maximum utilization potency of the strain, which used more resources for the purpose of acid production, improved resource utilization, and reduced the production cost of industrialized raw materials helpfully [12].

### 3.2 Exploring the optimal surfactant concentration through fermentation experiments

**Figure 2.**
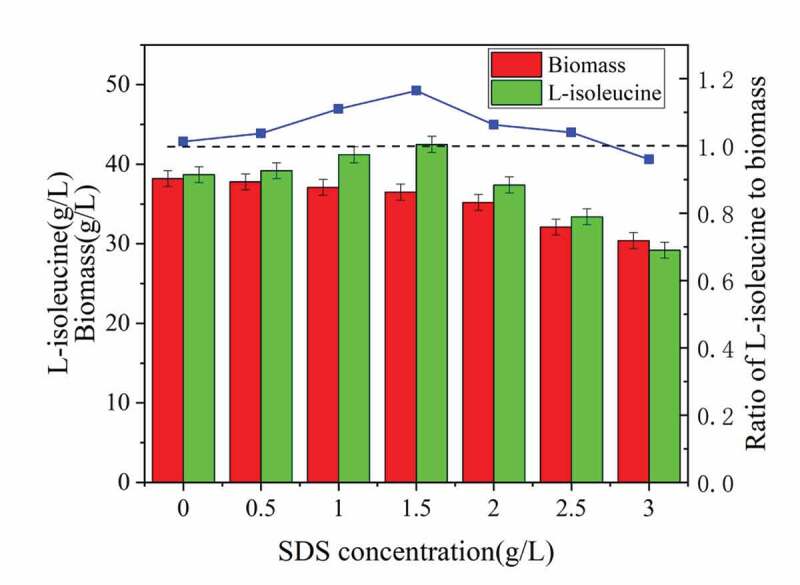
Effect of surfactant dosage on biomass and acid production of strain

The histogram of acid production and biomass in Figure 2 showed that different concentrations of SDS had an inhibitory effect on the biomass of the strain. As the SDS concentration increased, the damage to the strain increased. Elevated SDS concentration not only destroyed the cell membrane but also destroyed the cytoskeleton, released intracellular substances, and reduced the biomass., moreover, it also reduced the viability of the strain and the acid production capacity [13]. An extremely low SDS concentration had little effect on cell growth and metabolism, which not only cannot achieve the ability to change the permeability of the cell membrane, but also not significantly improved the ability to produce acid [14]. As seen in Figure 2, the addition of 1.5 g/LSDS in the initial stage of fermentation had the best effect. The strain biomass reached 36.5 g/L, which was 4.45% lower than that without SDS. However, the titer of L-isoleucine was 42.5. g/L, reduced by 9.81% compared with no SDS. In addition, the acid-to-mass ratio reached 1.16, which was 14.9% higher than without SDS, eventually leading to greatly improve the acid production capacity.

### 3.3. Effect of surfactant addition time on biomass and L-isoleucine

**Figure 3.**
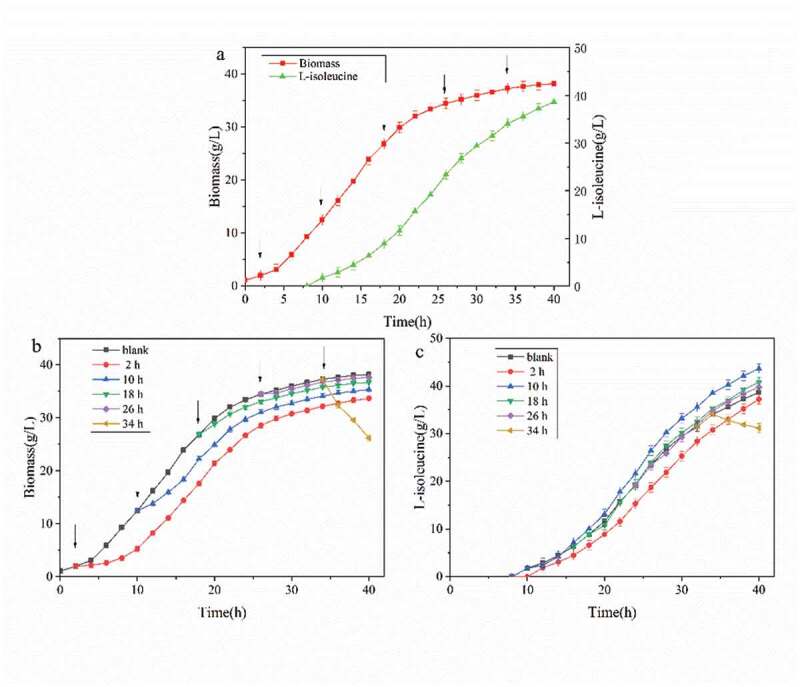
Effects of different adding time of SDS on strain biomass and acid production

In different growth stages, the morphology and metabolic pathway of the cell and the ability to respond to external stimuli were different. According to Figure 3(a), 1.5 g/L was added at different growth stages (2, 10, 18, 26, and 34 h) of the strain, and the effect of the strain on the adaptability and acid production ability of SDS was observed [15].

(a) SDS adds time; (b) Biomass process curve of SDS under different adding time; (c) The formation process curve of L-isoleucine under different adding time of SDS.

According to Figure 3(b), from 2 h to 26 h of fermentation, with the addition of the surfactant SDS, there was an adaptation period that was 2–4 h. At this time, the growth of the strain was slow or even stagnant, but the growth of the strain returned to normal after the adaptation period., in addition, the shorter the adaptation period led to a smaller impact on the growth of strain with a delay in SDS addition [16,17]. It can be seen that the biomass of cells decreased rapidly when 1.5 g/L SDS was added at 34 h of fermentation. The biomass of cells decreased from 37.21 g/g/L to 26.2 g/g/L within 6 h, and the lethal rate reached 29.6%. After 34 h of fermentation, the cell was in the decay stage, and the bacteriostatic factors and metabolites in the fermentation broth caused cell death [18]. Also, the addition of surfactant SDS destroyed the cell structure and accelerated cell fragmentation and autolysis [19].

As seen in Figure 3(c), when SDS was added at 10 h of fermentation, the acid production curve rose in the whole process after 4 h, and the acid production capacity was significantly improved compared with the control group. The final L-isoleucine production reached 43.67 g/L, which was 13.01% higher than that of the control group. At the initial stage of fermentation (2 h), the whole acid production capacity of SDS was lower than that without SDS, which means that adding surfactant early in the initial stage negatively affected acid production [20]. In the later stage of logarithmic growth (18 h) and stable period (26 h), the acid production capacity of SDS was lower than that without SDS. After adding SDS, the acid production curve had no obvious fluctuation, and the effect on the acid production capacity was not significant [21]. After adding SDS, the concentration of L-isoleucine in the fermentation broth decreased continuously, and the L-isoleucine concentration decreased by 8.5% at 40 h. The acid production rate was lower than the acid consumption rate of strain, and L-isoleucine showed an inverse growth. From the above experiments results, it can be seen that the addition of surfactants at different stages of the strain has a great impact on the growth and acid production capacity of the strain [22].

### 3.4 Effect of surfactant addition time on by-product formation and glucose conversion

**Figure 4.**
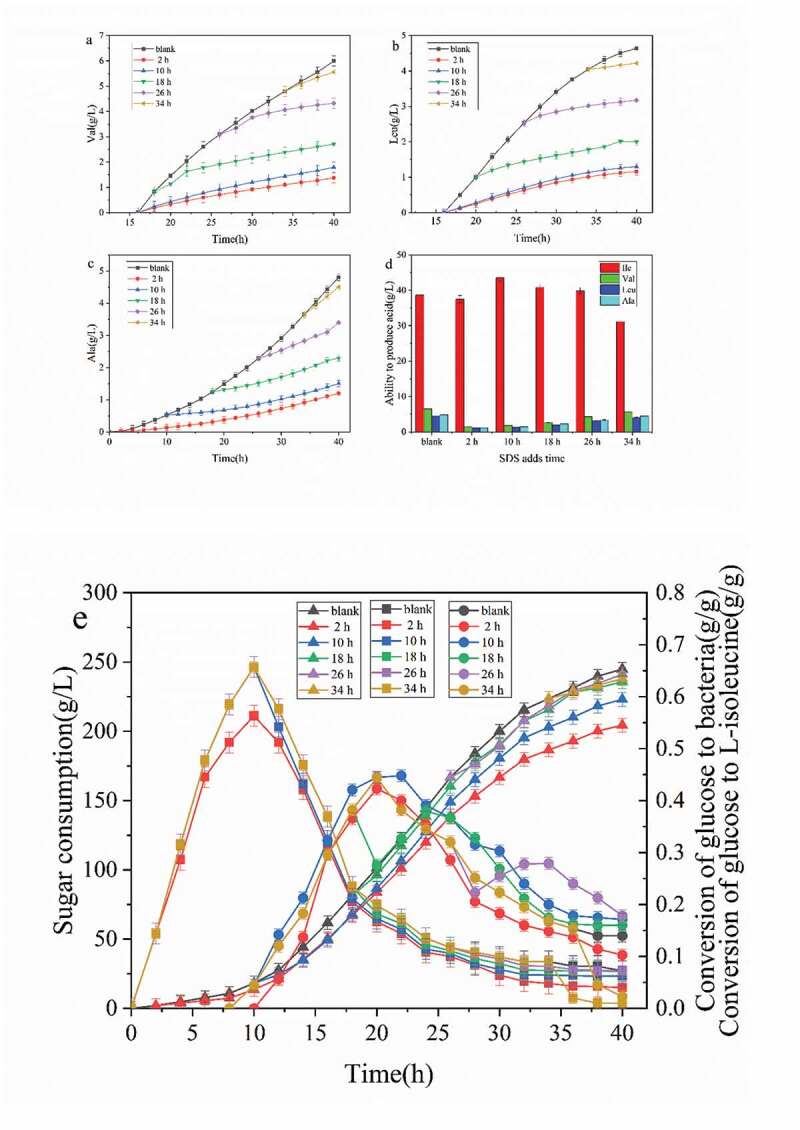
Effect of SDS addition time on fermentation parameters

As seen in Figure 4, the addition of surfactant SDS in the logarithmic growth period (10 h) increased L-isoleucine production and reduced by-products. After fermentation, L-isoleucine in the detection tank reached 43.5 g/g/L. The Val, Leu, and Ala by-products accumulated 1.8, 1.2, and 1.3 g/l, respectively, which were 72.3%, 64.3%, and 71.7% lower than those without SDS, respectively. The addition of SDS increased the permeability of the cell membrane, relieved the inhibition of intracellular L-isoleucine on precursor products, enhanced the main metabolic pathway, reduced its bypass metabolism, increased the production of L-isoleucine, and reduced the synthesis of by-products [23].

(a) Valine generation curve of SDS with different adding time; (b) Leucine generation curve of SDS with different adding time; (c) Alanine generation curve of SDS with different adding time; (d) Comparison diagram of main acid and accessory acid at different SDS adding time; (e) The process curve of sugar consumption and sugar acid conversion at different SDS adding time.

The sugar consumption curve (Figure 4) demonstrated that after adding SDS at any time of fermentation, the sugar consumption of strain decreased. With advanced SDS addition time, sugar consumption gradually decreased [24]. The curve of glucose conversion rate showed that delayed SDS addition in the fermentation process gradually tended to be the curve without surfactant, which showed that the influence of surfactant on the growth of strain gradually disappeared [25,26]. The addition of surfactant in the decay period (34 h) decreased the conversion curve of glucose on strain rapidly, and the cell growth rate decreased significantly, which was consistent with the growth curve in Figure 3(b). As shown in the curve of glucose to the L-isoleucine conversion rate curve, the addition of SDS strengthened the glucose synthesis pathway to L-isoleucine, and the conversion rate of L-isoleucine increased significantly by glucose [27]. The effect of adding SDS at 10 h was the most significant, and the conversion rate of glucose to L-isoleucine reached 0.392 at 22 h. The results showed that the conversion rate of L-isoleucine increased by 17.4% compared with that of the control group when SDS was added. The curve of the L-isoleucine conversion rate of glucose to L-isoleucine decreased sharply from the end of fermentation to 40 h, which was the same as that of the L-isoleucine curve.

### 3.5 Effect of surfactant addition on intracellular enzyme activity

**Figure 5.**
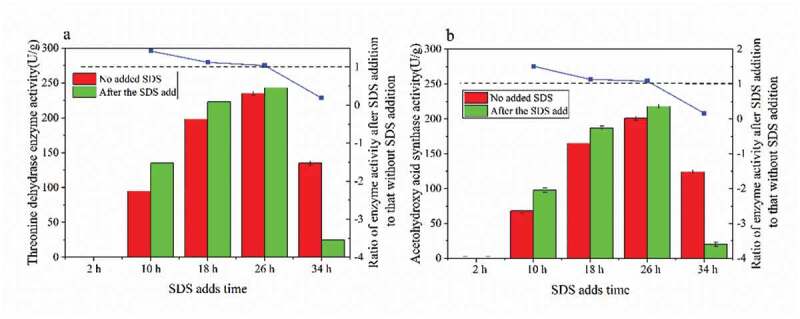
The activity of two rate-limiting enzymes from threonine to isoleucine

Threonine dehydratase is a type of isoleucine feedback-resistant enzyme. It catalyzes the degradation of threonine to α-ketobutyric acid, while acetylhydroxy acid synthase catalyzes α-ketobutyric acid. These two enzymes are the first and second reactions of the threonine to isoleucine pathway. As seen in Figure 5, the addition of surfactant SDS at 10 h had the greatest positive effect on the production of threonine dehydratase and acetylhydroxy acid synthetase. The activities of threonine dehydratase and acetylhydroxy acid synthetase were 135 and 98 (U/g), respectively. The activities of threonine dehydratase and acetylhydroxy acid synthase were the strongest at 26 h, but SDS had the least positive effect on the enzyme activity. In addition, there was no activity of threonine dehydratase and acetylhydroxy acid synthetase in the cells at 2 h. At this time, the addition of SDS had no effect on the production of L-isoleucine, but SDS had an inhibitory effect on the production of strain [28]. At the same time, it was found that after adding SDS at 34 h, the activities of both enzymes decreased, which had a negative effect on the production of L-isoleucine.

(a) Threonine dehydrase enzyme activity; (b) Acetohydroxy acid synthase enzyme activity.

### 3.6 Metabolic flow analysis of the effect of surfactants on L-isoleucine production

**Figure 6.**
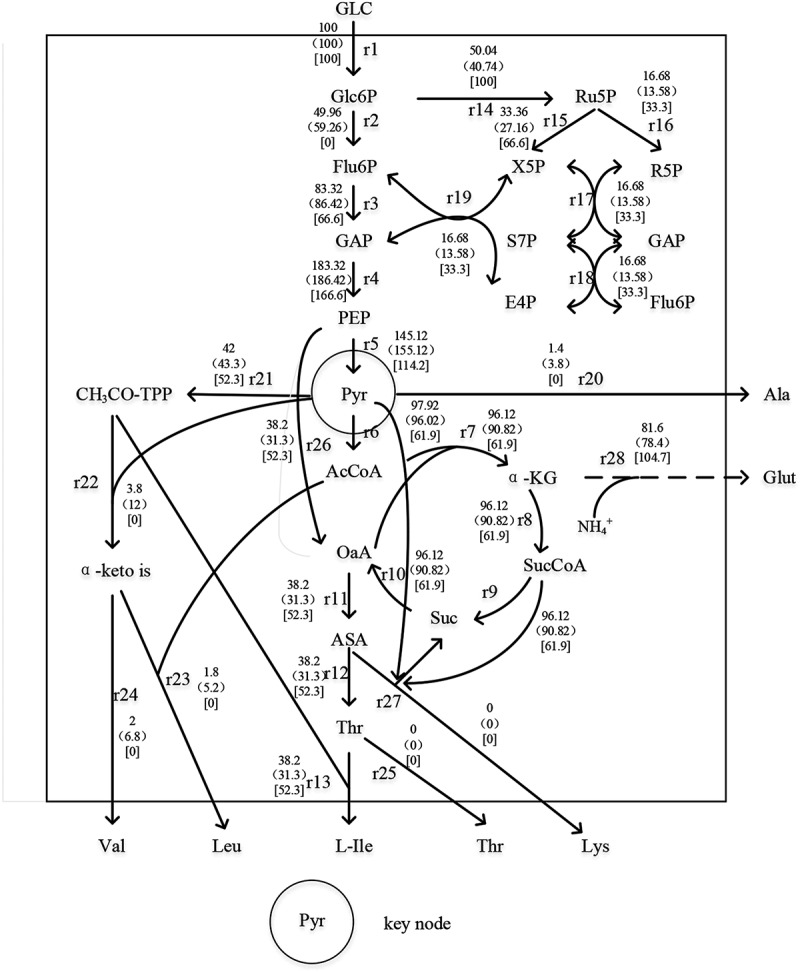
L-isoleucine metabolic flow node analysis

In this study, SDS was added as the experimental group and SDS without surfactant as the control group. The changes in intracellular metabolic nodes were explored by the metabolic flow chart, as shown in Figure 6.

According to the steady-state hypothesis, the synthesis rate of the intermediate product is equal to its metabolic rate. According to this condition, the equilibrium equation was established. As seen in Table 1. The number of equations in the table is 21, the unknown number is 28, and the degree of freedom of the equation is 7.

**Table 1.** Equilibrium equation

Intermediate products	Equilibrium equation
G6P	r1-r2-r14 = 0
Ribu5P	r14-r15-r16 = 0
Xyl5P	r15-r19-r17 = 0
Rib5P	r16-r17 = 0
E4P	r19-r18 = 0
Sed7P	r17-r18 = 0
Fru6P	r2-r3+ r19+ r18 = 0
GAP	2r3-r4+ r19+ r17-r18 = 0
PEP	r4-r5-r26 = 0
Pyr	r5-r6-r22-r21-r27-r20 = 0
AcCoA	r6-r23-r7 = 0
ɑ-KG	r7-r8 = 0
Glut	r28-r11-r27-r13-r20-r24-r23 = 0
SucCoA	r8-r9-r27
Suc	r9-r10+ r27 = 0
OAA	r10-r7-r11+ r26 = 0
ASA	r11-r12-r27 = 0
Thr	r12-r25-r13 = 0
Ac-TPP	r21-r22-r13 = 0
ɑ-keto is	r22-r24-r23 = 0
NADPH	2r14+ r1-r28-r11-r27-r12-r13 = 0

#### 3.6.1 Glucose-6-phosphate nodal metabolic flow analysis

Glucose has three main metabolic pathways, namely EMP, HMP, and TCA. The metabolic flow of glucose into the EMP pathway was 49.96, which was 15.69% lower than that of the control group. Also, the metabolic flow of glucose into the HMP pathway was 50.04, which was 22.83% higher than that of the control group, and was 50.04% of the theoretical maximum value. The ratio of glucose metabolic flows through EMP to HMP was approximately 1, it can be seen that the addition of surfactant SDS could changed the fermentation conditions. Some experimental results showed that the pentose phosphate produced by HMP is all converted into intermediate products in the EMP pathway, and at the same time provides a large amount of reducing power NADPH for the synthesis of amino acids. Therefore, increasing the ratio of metabolic fluxes of HMP and EMP is beneficial to the synthesis of amino acids [29].

#### 3.6.2 Nodal metabolic flow analysis of pyruvate and phosphoenolpyruvate

Phosphoenolpyruvate (PEP) generates oxaloacetic acid, which is the precursor for synthesizing other amino acids, through a CO_2_ fixation reaction. In the Figure 6, 38.2 PEP forms oxaloacetic acid through a CO_2_ fixation reaction, so it is necessary to enhance the replenishment pathway of the TCA cycle to enhance the synthesis of Ile. It can be seen that the flow of PEP to PYR decreased by 6.45% compared with the control group. However, pyruvate (PYR) is the direct precursor of Val, and Leu is synthesized by the branching of α-acetylisovaleric acid of the intermediate of the Val synthesis pathway. Therefore, the production of valine and leucine can be reduced by limiting the production of pyruvate[1].

The metabolic flow from PYR to Ala was 1.4, which was 63.16% lower than that of 3.8 in the control group. Because the strain was Lys deficient, there was no Lys accumulation. According to the analysis of PYR and PEP nodes, it was necessary to strengthen the replenishment path of the TCA cycle, weaken the flow of PEP into the TCA cycle, reduce energy production, inhibit the growth of strain to reduce the production of Ala.

#### 3.6.3 Analysis of Thr node metabolic flow

As the direct precursor of Ile, the metabolic flow of aspartic acid (ASA) into Thr in the experimental group was increased by 22.04% compared with that in the control group, and all of them flowed into the formation of Ile without Thr outflow. The enhanced replenishment pathway of the TCA cycle directly led to the generation of ASA in the downstream pathway, which was conducive to the accumulation of Ile [30].

#### 3.6.4 Nodal metabolic flow analysis of ASA

Because four of the six carbon atoms of Ile come from ASP, and Ile, together with Thr, Lys, and Met, are called ASP group amino acids, it is necessary to enhance the source of ASP and ASA [31]. The metabolic flow from oxaloacetic acid to ASA was 38.2, which was 22.04% higher than that of the control group. All of them flowed to Thr synthesis, and Lys accumulation was not found.

#### 3.6.5 Node metabolic flow analysis of α-keto iso

According to the metabolic map, α-keto iso is the direct precursor of both valine and leucine, which can reduce the accumulation of valine and leucine by weakening the source of synthesis [32]. In this study, the metabolic flow of CH_3_CO-TPP into α-keto iso was 3.8, which was 69.25% lower than that in the control group. The metabolic flow into Val was decreased by 68.33%, and the metabolic flow of Leu synthesis decreased by 65.38%. The main by-products Val and Leu were 0 in the ideal metabolic flow distribution, indicating that Val and Leu could be completely absent in the whole metabolic process.

## 4 Conclusion

In the process of producing L-isoleucine by *C. glutamicum*, the chemical means of adding surfactant SDS can interfere or destroy the synthesis of the cell membrane or the cell membrane itself, which can realize the ‘fermentation conversion’ faster. The yield of L-isoleucine reached 43.67 g/L after adding 1.5 g/L surfactant SDS at 10 h of fermentation. Val, Leu, and Ala were reduced by 72.3%, 64.3%, and 71.7%, respectively. The addition of SDS increased the permeability of the cell membrane, relieved the inhibition of intracellular L-isoleucine on precursor products, enhanced the main metabolic pathway, reduced its bypass metabolism, increased the production of L-isoleucine, and reduced the synthesis of by-products.

## Highlights

- Effects of surfactants on biomass and acid production of *C. glutamicum* were studied.
- Surfactants improved the permeability of *C. glutamicum* cell membranes.
- L-isoleucine yield increased by 13.01%.
- Val, Leu, and Ala yield decreased by 72.3%, 64.3%, and 71.7%, respectively.
- It was confirmed that the surfactant changed the intracellular metabolic flux.
